# Whole exome sequencing identified a rare *WT1* loss‐of‐function variant in a non‐syndromic POI patient

**DOI:** 10.1002/mgg3.1820

**Published:** 2021-11-29

**Authors:** Yingchen Wang, Qing Chen, Feng Zhang, Xi Yang, Lingyue Shang, Shuting Ren, Yuncheng Pan, Zixue Zhou, Guoqing Li, Yunzheng Fang, Li Jin, Yanhua Wu, Xiaojin Zhang

**Affiliations:** ^1^ Obstetrics and Gynecology Hospital NHC Key Laboratory of Reproduction Regulation (Shanghai Institute of Planned Parenthood Research) School of Life Sciences Fudan University Shanghai China; ^2^ Institute of Metabolism and Integrative Biology Fudan University Shanghai China; ^3^ Shanghai Key Laboratory of Female Reproductive Endocrine Related Diseases Shanghai China; ^4^ National Demonstration Center for Experimental Biology Education School of Life Sciences Fudan University Shanghai China

**Keywords:** premature ovarian insufficiency (POI), truncated protein, whole exome sequencing (WES), Wilms’ tumor, WT1

## Abstract

**Background:**

Premature ovarian insufficiency (POI) is a highly heterogeneous disease, and up to 25% of cases can be explained by genetic causes. The transcription factor *WT1* has long been reported to play a crucial role in ovary function. *Wt1*‐mutated female mice exhibited POI‐like phenotypes.

**Methods and Results:**

In this study, whole exome sequencing (WES) was applied to find the cause of POI in Han Chinese women. A nonsense variant in the *WT1* gene: NM_024426.6:c.1387C>T(p.R463*) was identified in a non‐syndromic POI woman. The variant is a heterozygous de novo mutation that is very rare in the human population. The son of the patient inherited the mutation and developed Wilms’ tumor and urethral malformation at the age of 7. According to the American College of Medical Genetics and Genomics and the Association for Molecular Pathology (ACMG/AMP) guidelines, the novel variant is categorized as pathogenic. Western blot analysis further demonstrated that the *WT1* variant could produce a truncated WT1 isoform in vitro.

**Conclusions:**

A rare heterozygous nonsense *WT1* mutant is associated with non‐syndromic POI and Wilms’ tumor. Our finding characterized another pathogenic *WT1* variant, providing insight into genetic counseling.

## Graphical abstract

A heterozygous nonsense WT1 mutant is associated with non‐syndromic POI and Wilms’ tumor. The variant is a very rare mutation in the human population and is categorized as pathogenic. The nonsense variant caused a truncated protein product in vitro.

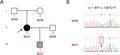

## 1 INTRODUCTION

Premature ovarian insufficiency (POI) is defined as absent menarche or premature depletion of ovarian follicles before the age of 40 years (Persani et al., 2009). POI is an extremely heterogeneous disorder with variable clinical presentations and multiple causes. It is estimated that genetic causes account for approximately 20%–25% of cases of POI (Jiao et al., 2018). In the past decade, multiple genetic analysis methods including whole exome sequencing (WES) have offered great opportunities to identify pathogenic variants in POI. POI‐associated causative genes fall within pathways critical for ovarian development and function, such as DNA damage repair, meiosis, recombination, gene transcription or translation, follicle development, steroidogenesis, etc. (Jiao et al., 2020; Rossetti et al., 2017).

The human *WT1* gene (OMIM 607102), located at 11p13 (GRCh37), encodes a transcription factor involved in transcriptional regulation, self‐association, and RNA recognition (Kennedy et al., 1996; Moffett et al., 1995; Reddy et al., 1995; Rose et al., 1990). Initially, *WT1* was found to be expressed at a high level in the glomeruli of the kidney and was first known as a tumor suppressor gene for Wilms’ tumor in the 1990s (Haber et al., 1990; Pelletier, Schalling, et al., 1991). WT1 protein contains a proline/glutamine‐rich domain at the N‐terminus and four zinc fingers in the C‐terminal region (Bardeesy & Pelletier, 1998). A repression domain is located within residues 84–179, and an activation domain with independent function is between residues 180 and 294 (Wang et al., 1993). WT1 binds to DNA helix through the four carboxyl‐terminal Cys2His2 zinc fingers, which have bidirectional activities of transcriptional regulation depending on the cellular or chromosomal context (Parenti et al., 2015; Ullmark et al., 2018).

To date, *WT1* is found to be expressed and functional in many tissues, with essential roles in the regulation of ovarian cell proliferation, apoptosis, and steroidogenesis (Park et al., 2014; Pelletier, Bruening, et al., 1991; Wang et al., 2021). For instance, activation of *WT1* through the regulation of the upstream activator *Bax* is necessary for the maintenance of granulosa cell survival during the early stage of follicles in rats (Park et al., 2014). Multiple steroidogenic enzyme‐encoding genes have also been reported to be putative targets of *WT1*. In mouse ovaries, the mRNA levels of *P450scc*, *3β*‐*HSD*, *Hsd17b1*, *Cyp17a1*, *Star*, and *Arx* were significantly increased in *Wt1*‐deficient XX gonads compared with those in control ovaries (Chen et al., 2017). Moreover, variants in the *Wt1* gene in animals are associated with ovarian insufficiency. Severe reproductive defects such as smaller ovaries and reduced number of follicles were observed in *Wt1* ^+/R394W^ female mice (Gao et al., 2004).

Here, we identified a nonsense variant of *WT1* in a non‐syndromic POI patient and her son from a non‐consanguineous Chinese family through WES data processing. Human genome variation databases were utilized to investigate the minor allele frequency, and bioinformatic tools were utilized to evaluate the pathogenicity. Sanger sequencing was performed on the patient and her family members to confirm their genotypes. The western blot assay suggested that the *WT1* variant could encode a truncated protein, which might contribute to the development of POI.

## 2 MATERIALS AND METHODS

### 2.1 Study subject and clinical evaluations

POI patients were diagnosed at the Affiliated Obstetrics and Gynecology Hospital of Fudan University. The criteria for POI diagnosis follow the recommendations provided by the European Society for Human Reproduction and Embryology (2016) (European Society for Human Reproduction and Embryology (ESHRE) Guideline Group on POI et al., 2016). Women with ovarian surgery and radiotherapeutic or chemotherapeutic interventions were excluded. A detailed clinical query including environment, behavior, diet, and poison exposure was also performed. Familial history was ascertained. Written informed consent was obtained from participants or the parent of participants under the age of 18.

### 2.2 DNA extraction and assessment

Genomic DNA was extracted from peripheral blood using the QIAamp DNA Mini Kit (QIAGEN, Hilden, Germany) according to the manufacturer's instructions. Briefly, optimized buffers and enzymes were used to lyse the peripheral blood from the patient, stabilize nucleic acids, and enhance the genome DNA adsorption to the QIAamp membrane. Then, alcohol was added, and the whole lysates were loaded onto the QIAamp spin column. Afterward, wash buffers were used to remove impurities, and pure ready‐to‐use DNA was then eluted in water or a low‐salt buffer. Finally, the quality and quantity of DNA were assessed by agarose gel electrophoresis and SimpliNano (Harvard Bioscience).

### 2.3 WES and data processing

Approximately 1.5 μg of genomic DNA was used to prepare a captured library using an Agilent SureSelectXT Human All Exon V6 kit and then sequenced on a HiSeq X Ten platform (Illumina). Raw data were aligned to the human reference genome sequence (UCSC Genome Browser hg19) with the Burrows‐Wheeler Alignment tool (http://bio‐bwa.sourceforge.net/). Variant calling was accomplished using the Genome Analysis Toolkit (https://www.broadinstitute.org/gatk/) (McKenna et al., 2010) and ANNOVAR software was used to annotate all variants.

The raw data collected from WES were subjected to analysis as previously described (Yang et al., 2019). Briefly, genetic variants in the exonic and splicing regions were chosen. Variant filtering was performed based on a minor allele frequency (MAF) ≤0.1% in the 1000 Genomes Project (1KG Project; http://browser.1000genomes.org), Genome Aggregation Database (gnomAD; http://gnomad‐old.broadinstitute.org/), and Exome Aggregation Consortium (ExAC; http://exac.broadinstitute.org). Predictions of deleterious nonsynonymous variants were performed using four bioinformatics tools: SIFT (http://sift.jcvi.org), PolyPhen‐2 (http://genetics.bwh.harvard.edu/pph2/), MutationTaster (http://www.mutationtaster.org), and CADD (http://cadd.gs.washington.edu).

### 2.4 Variant confirmation

Sanger sequencing was performed to confirm the potential causative variants in the family. Genomic DNA was used for variant confirmation. Primers for the *WT1* (NM_024426.6) variant were designed using the “Primer‐BLAST” program (https://www.ncbi.nlm.nih.gov/tools/primer‐blast/). Primer specificity was checked using the alignment search tool BLAST (https://www.ncbi.nlm.nih.gov/blast).

**FIGURE 1:**
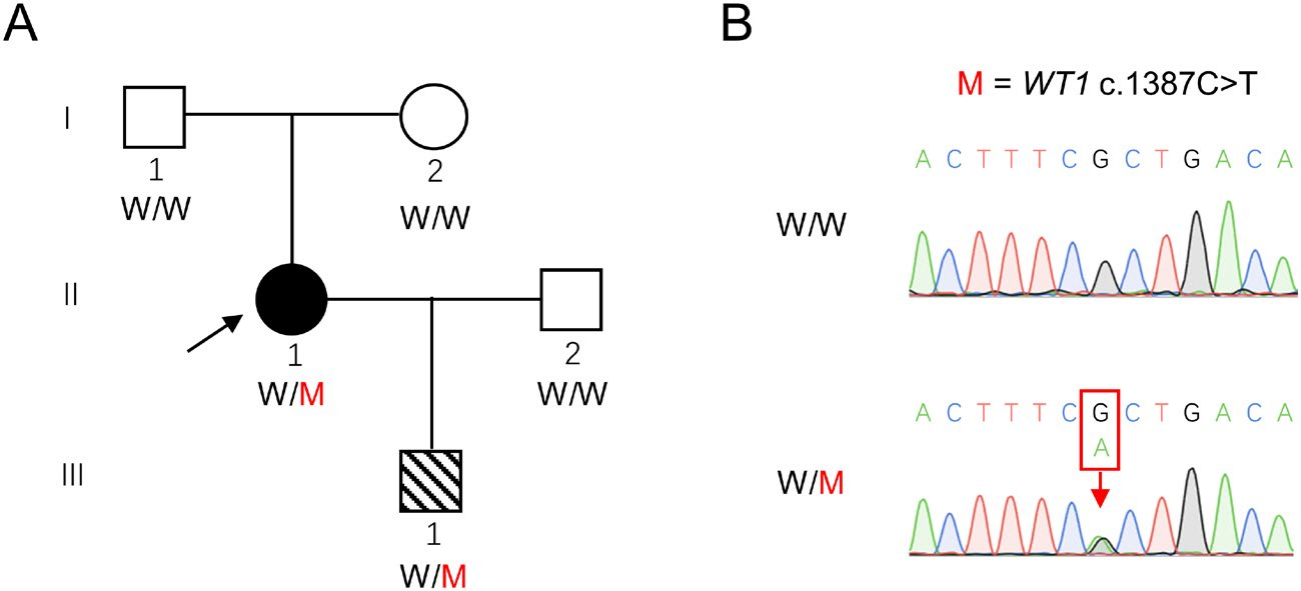
Identification of a *WT1* mutation in a Chinese family. (a) A heterozygous *WT1* variant (M) was identified in a non‐consanguineous family. The black arrow in the pedigree plot indicates the proband. (b) Sanger sequencing confirmed heterozygous *WT1* mutations in the proband (W/M). Both of the proband's father and mother are wild type (W/W). The red arrow indicates the mutation sites

Primer sequences were as follows: forward, 5′–GGAA ACAGTAGGGACCTGGC‐3′; reverse, 5′–CAGATGCAGAC ATTGCAGGC‐3′. The results of Sanger sequencing were analyzed using SnapGene 4.2.4 software (Figure 1).

### 2.5 Plasmid construction and mutagenesis

Full‐length human *WT1* cDNA was synthesized (Weizhen, Jinan, China) and constructed into the pCMV‐FLAG vector (Takara). Site‐directed mutagenesis was performed to generate the null variant (c.1387C>T) of *WT1* according to the instructions of the KOD‐Plus‐Mutagenesis Kit (Toyobo). The relevant primers were as follows: forward, 5′‐TGAAAGTTCTCCCGGTCCGACCACC‐3′; reverse, 5′‐CTGACAAGTTTTACACTGGAATGGTTTCACACCTGT‐3′. The recombinant plasmids were verified by direct Sanger sequencing prior to functional studies.

### 2.6 Cell culture and transfection

Human embryonic kidney 293T (HEK293T) cells were purchased from the Cell Bank of the Chinese Academy of Sciences. HEK293T cells were cultured in Dulbecco's Modified Eagle's Medium (DMEM) (Gibco) supplemented with 10% fetal bovine serum (FBS) (Gibco) and 1% penicillin–streptomycin–neomycin (PSN) antibiotic mixture (Gibco) at 37℃ with 5% CO_2_. HEK293T cells were transfected with the wild‐type or mutated *WT1* plasmids using Lipofectamine 3000 (Invitrogen) according to the manufacturer's instructions.

### 2.7 Western blotting

Whole cell lysates were separated by SDS‐PAGE and transferred onto PVDF membranes. After being blocked with nonfat milk, each membrane was incubated with specific antibodies against different proteins at 4℃ overnight, followed by incubation with an HRP‐conjugated secondary antibody. Membranes were visualized using an enhanced chemiluminescence kit (GE Healthcare Life Science). The images acquired were representative of three independent experiments with consistent results. β‐actin was used as a loading control. The related antibodies included anti‐FLAG (cat. no. F3165, Sigma‐Aldrich), anti‐GFP (cat. no. G6539, Sigma‐Aldrich), HRP‐labeled anti‐β‐actin (cat. no. HRP–60008, Proteintech), HRP‐labeled goat anti‐mouse IgG (cat. no. I‐0031, DingGuo Changsheng Biotech), and HRP‐labeled goat anti‐rabbit IgG (cat. no. IH‐0011, DingGuo Changsheng Biotech).

## 3 RESULT

### 3.1 Clinical findings

The diagnosis of POI is based on the presence of menstrual disturbance and biochemical confirmation, in brief: (i) oligo/amenorrhea for at least 4 months; (ii) an elevated FSH level >25 mIU/ml on two occasions >4 weeks aside; (iii) no fallopian tube abnormalities; (iv) no radioactive, surgical, or chemotherapeutic injury; (v) no inflammation or autoimmune response of the pelvic cavity or reproductive system; and (vi) no karyotypic abnormality.

**FIGURE 2:**
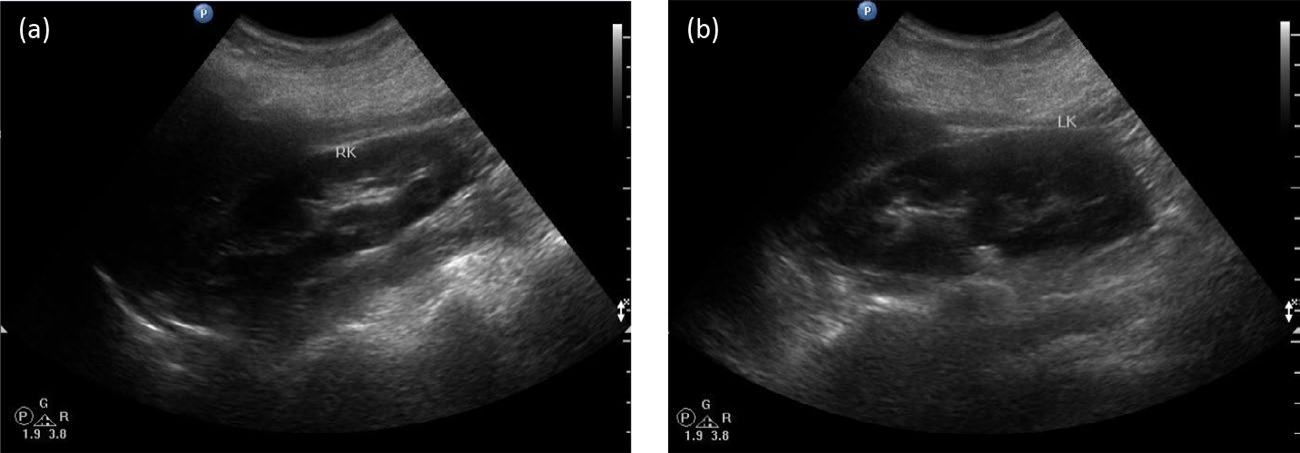
Transabdominal ultrasound image of the POI subject. Ultrasound of the right kidney (a) and left kidney (b) from the proband showed normal size, structure and position of kidneys and ureters

**FIGURE 3:**
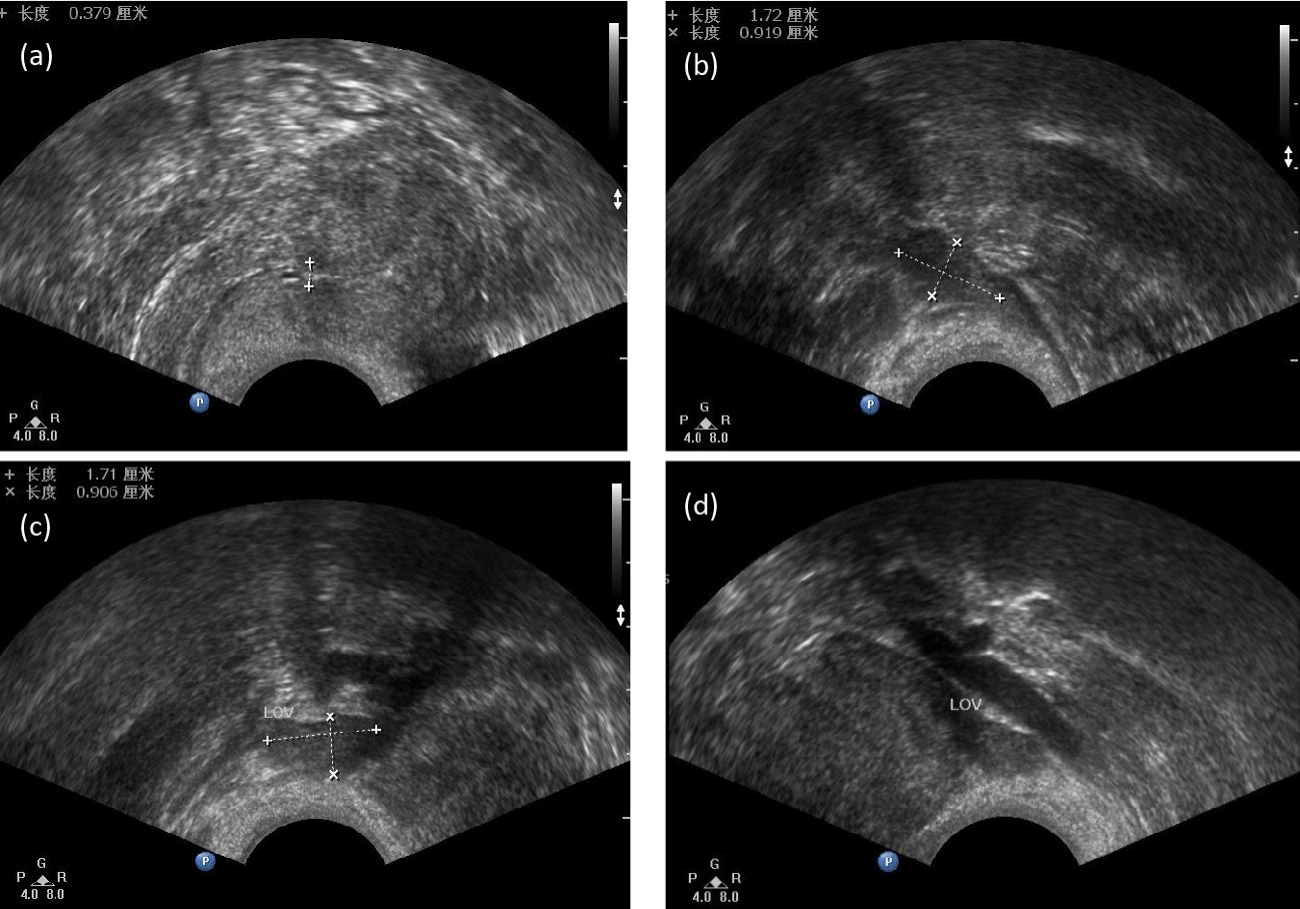
Transvaginal ultrasound image of the POI subject. (a) The thickness of endometrium. (b) Size of the right ovary. (c and d) Size of the left ovary

As shown in Figure 1a, a 26‐year‐old woman (II–1) diagnosed with POI from a Chinese Han non‐consanguineous family was ascertained in this study (Figure 1a). The proband had normal puberty, and menarche occurred at 16 years of age. Her menses became irregular at 23 years of age and completely stopped at 26 years of age. Other probable histories including ovarian operation, chemotherapy, radiotherapy, or immune disease were all excluded. Physical examination showed a normal body mass index. No other known urologic diseases (Table S1; Figure 2), endocrinopathies, or autoimmune disorders (Table S2) were observed for the proband. Transvaginal ultrasonography revealed a normal uterus but small ovaries with few antral follicles (Figure 3). Consecutive hormonal measurements revealed elevated FSH levels. Clinical information regarding the POI subjects is summarized in Table 1.

**TABLE 1:** Clinical characteristics of the POI patient affected by *WT1* variant

Characteristic	Proband
First menses (y old)	16
Age of POI (y old)	26
Weight (kg)	53
Height (cm)	158
FSH (mIU/ml)	67.84
LH (mIU/ml)	49.4
PRL (ng/ml)	11.44
E2 (pg/ml)	71
P (pg/ml)	0.5
T (ng/ml)	0.43
Size of ovary (right/left) (mm)	16 × 13 × 10/25 × 23 × 17
Size of follicle (right/left) (mm)	Not detected/19 × 16 × 14

Abbreviations: E2, estradiol; FSH, follicle‐stimulating hormone; LH, luteinizing hormone; P, progesterone; PRL, prolactin; T, testosterone.

### 3.2 Identification of a rare *WT1* variant by WES

WES was performed on peripheral blood DNA from the patient. Analysis of WES data was performed as previously described (Yang et al., 2019). Filtering steps and variants identified in each step are shown in Table 2. Among all variants called by WES, 11,633 variants of high calling quality and sited in exonic and splicing regions were reserved. Variants with a MAF of more than 0.1% were then excluded according to three public human genome variation databases (1KG Project, ExAC, and gnomAD). Then, 195 synonymous variants were further excluded. Among the 502 remaining variants, 321 were missense, which was subjected to functional prediction using in silico tools. Relevance to phenotype was considered based on previous reports and animal studies. Finally, a heterozygous variant of *WT1*, NM_024426.6:c.1387C>T (p.R463*; rs121907909) was identified. This was confirmed by Sanger sequencing (Figure 1). As shown in Table 3, the allele frequency of *WT1* c.1387C>T in total population is 0.000006583 (1/151,896), and the only case is a European male. It is predicted to be pathogenic by DANN, MutationTaster, and CADD.

**TABLE 2:** Filtering steps and variants identified in each step

Step	Number of variants
All variants called by WES	43362
High calling quality	38783
In exonic and splicing regions	11633
Allele frequencies ≤0.001 in databases^a^	697
After elimination of synonymous SNVs	502
Nonsense, frameshift, non‐frameshift indel, splicing site, or deleterious missense variants^b^	243
Known pathogenic genes of POI	1

^a^ Allele frequencies were estimated according to 1KG Project, ExAC, and gnomAD databases.

^b^ All missense variants were assessed using the SIFT, PolyPhen‐2, MutationTaster, and CADD tools. From those, deleterious variants were selected.

**TABLE 3:** In silico analysis of identified variant in *WT1* gene

Gene	Mutation type	cDNA Change^a^	Protein change	Minor allele frequency^b^			Functional prediction^c^		
				1KG	ExAC	gnomAD	DANN	MutationTaster	CADD
*WT1*	Heterozygous	c. C1387T	p.R463*	0	0	0.000006583	Damaging	Damaging	14.003

^a^ The GenBank accession number of *WT1* is NM_024426.4.

^b^ Allele frequencies were estimated according to the 1KG Project, ExAC, and gnomAD databases.

^c^ Mutation assessment using MutationTaster and CADD tools. High CADD scores suggest that a variant is likely to have deleterious effects. The CADD cutoff is usually set at 4.

### 3.3 Family follow‐up and genetical analysis

The proband's parents were both healthy without any other diseases. Her mother (I–2) was now 51 years old and still experiencing a regular period. She also denied a history of any reproductive and urological diseases. Sanger sequencing revealed that both parents of the proband were wild type. Therefore, *WT1* c.1387C>T is a de novo variant for the proband. Classification of the variant was then performed according to the ACMG/AMP guidelines and this novel variant was classified as “pathogenic.”

Additionally, the proband has one son (III–1) and he has been diagnosed with Wilms’ tumor and urethral malformation at 7 years of age. Sanger sequencing demonstrated that he inherited the mutant *WT1* variant from his mother and a wild‐type *WT1* allele from his father, so his genotype was the same as his mother.

### 3.4 In vitro functional characteristics of the *WT1* variants

**FIGURE 4:**
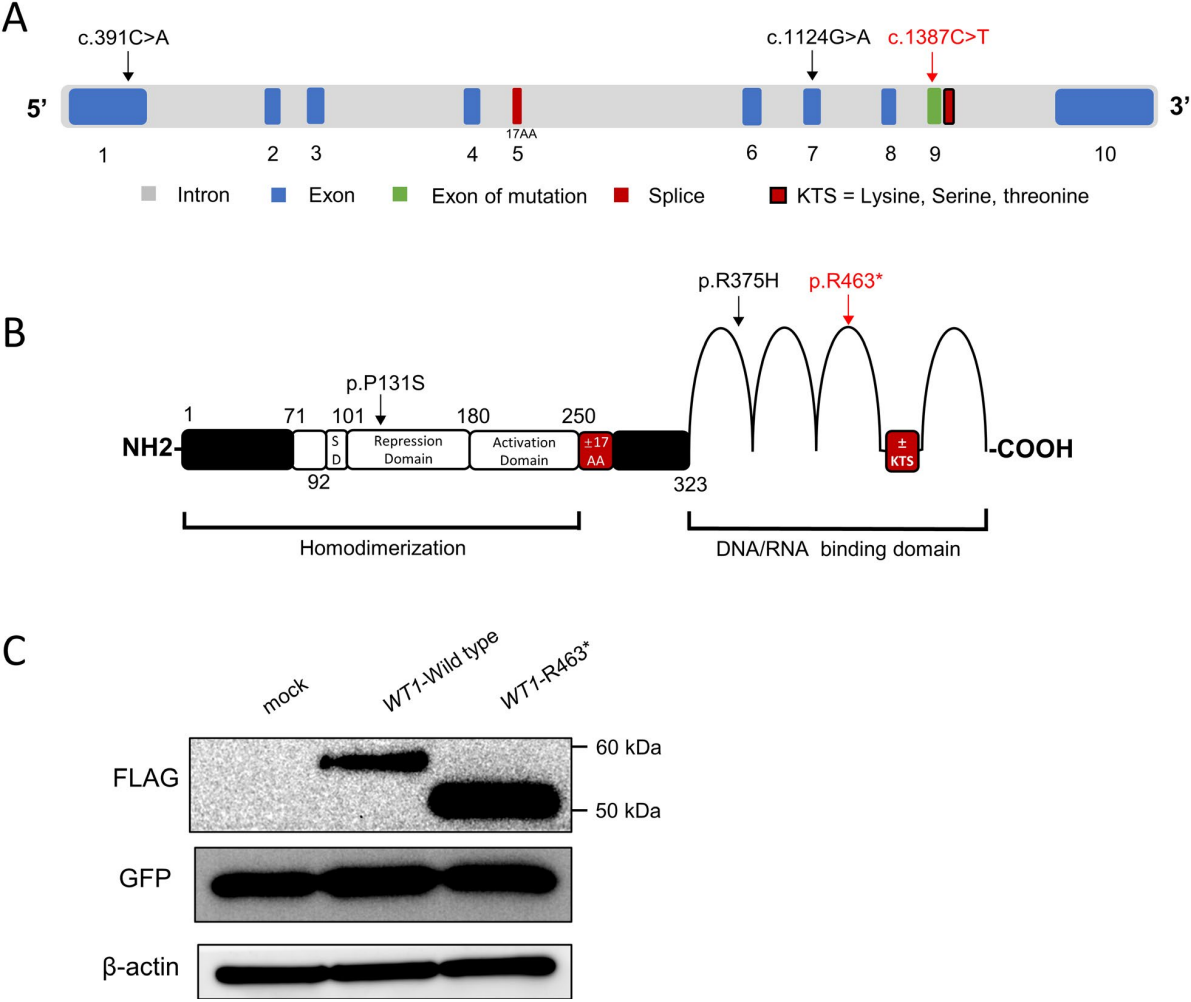
Schematic representation of the *WT1* gene and protein. (a) *WT1* is comprised of 10 exons. Two alternative splicing sites are indicated by red boxes. The black arrows indicate two mutations related to POI from previous report, while the red arrow indicates the variant we found. (b) Known functional domains of WT1 protein include the homodimerization domain and DNA/RNA‐binding domain (zinc finger domain). The inclusion of exon 5 leads to the insertion of 17 amino acid residues into the regulatory domain of WT1, which is indicated as “±17AA” in the red box. Four arcs represent the zinc finger domain. The alternative splicing at the end of exon 9 produced the tripeptide KTS, which is inserted between zinc fingers III and IV and indicated by a red box. The variant reported by us is marked by a red arrow, and two mutations from previous report are marked by black arrows. (c) A truncated WT1 protein with approximately 51 kDa caused by the WT1 variant. Western blotting analysis of the WT1 protein expression in HEK293T cells transfected with equal amounts of indicated *WT1* constructs. GFP was used to evaluate the transfection efficiency and β‐actin was used as a loading control

*WT1* c.1387C>T variant was located in the ninth exon of *WT1*, and it introduced a premature stop codon in the second zinc finger of WT1 (NP_077744.4) (Figure 4a,b). The putative impact of the nonsense variant on WT1 was further investigated in vitro. Recombinant plasmids of full‐length wild‐type and mutated human *WT1* were introduced into HEK293T cells, respectively. The western blotting analysis revealed that a truncated protein of approximately 51 kDa in cells overexpressing the mutated *WT1* (Figure 4c), which was consistent with our prediction.

## 4 DISCUSSION

WT1 is a vital factor in maintaining female gonad development (Kreidberg et al., 1993). To date, a few studies have focused on how WT1 functions in regulating gonad development and female fertility using genetically modified animals. Herein, we summarized the female reproductive phenotypes from representative mouse models carrying different *Wt1* variants (Table 4). The targeted total deletion of the *Wt1* gene produced mice displaying hermaphroditism or gonadal dysgenesis, while heterozygous loss induced similar but much milder gonadal developmental defects, irrespective of the strain. Furthermore, mice with different mutation types differ in manifestations. Some showed masculinization with normal fertility while others had POI‐like phenotypes, indicating that characterization of different *WT1* variants is important in genetic analysis of females with ovarian dysfunction.

**TABLE 4:** Representative studies of *Wt1* genetically modified mice with ovarian defects

Index	Strain	Type	Phenotype of ovary	Ref
1	C57BL/6	*Wt1^−/−^*	Complete agenesis of the gonads.	Kreidberg et al. (1993)
		*Wt1^+/−^*	Normal in gonad	
2	C57BL/6×129/Sv	*Wt1^+/−^*	Smaller ovaries with fewer ova; normal appearance and maintenance of corpus lutea; no implanted embryos	Kreidberg et al. (1999)
3	129S7/SvEvBrd×C57BL/6J	*Wt1^tm1Asc/tm1Asc^*	Germ cells are fewer and abnormally organized; Gonads of XY mice are ovarian‐like and cryptorchid	Hammes et al. (2001)
		*Wt1^+/tm1Asc^*	Same as homozygous	
4	129S7/SvEvBrd×C57BL/6J	*Wt1^tm2Asc/tm2Asc^*	Streak gonad found in both XX and XY genotypes and obvious by E12.5; abnormal internal genital duct development	Hammes et al. (2001)
5	129P2/OlaHsd×C57BL/6	*Wt1^tm1Mlh/tm1Mlh^*	Agonadal (ovary absent in all E13.5 embryos) in embryos	Patek et al. (2008)
		*Wt1^+/tm1Mlh^*	Infertile	
6	B6/129	*Wt1^+/R394W^*	Subfertile; ovulation rate significantly decreased; ovaries significantly smaller; total number of developing follicles significantly reduced	Gao et al. (2014)
7	NA	*Wt1^+/−^*	Ectopic development of 3β‐HSD‐positive steroidogenic cells; aberrant differentiation of somatic cells in *Wt1*‐deficient gonads; *SF1* expression was dramatically upregulated in *Wt1*‐deficient XX gonads	Chen et al. (2017)
8	C57BL/6	*Wt1^+/−^*	Loss of sex‐specific gene expression pattern; reduced proliferating cells in XX gonad/mesonephroi explants	Rudigier et al. (2017)
9	129/SvEv×C57BL/6	*Wt1^+/−^*	Aberrant ovary development; pre‐granulosa cells to steroidogenic cells transformation; delayed meiosis progression in germ cells; abnormal degeneration of wolffian duct in *Wt1*‐deficient female embryos	Cen et al. (2020)
10	NA	*Wt1^+/R495G^*	Normal and fully fertile	Eozenou et al. (2020)
		*Wt1^R495G/R495G^*	Distinct signs of masculinization	

Genetic variation is one of the main causes of POI (Jiao et al., 2018; Persani et al., 2010; Veitia, 2020). In our study, we identified a de novo nonsense variant of the *WT1* gene in a non‐syndromic POI patient through WES. Wang et al. have previously identified two novel missense mutations and four intronic variants of *WT1* in 384 Chinese POI women (Wang et al., 2015). Mutations in *WT1* can cause many different diseases including non‐syndromic POI and syndromic POI such as Denys–Drash syndrome (Wang et al., 2018), WAGR syndrome (Huynh et al., 2017), and Frasier syndrome (Barbaux et al., 1997; Klamt et al., 1998). For our proband, the heterozygous *WT1* c.1387C>T variant caused non‐syndromic POI. As to the son, the renal cells might receive a second hit in its remaining functional copy of *WT1*, leading to the development of Wilms’ tumor (Cresswell et al., 2016).

*WT1* c.1387C>T has been reported in several patients with Wilms’ tumor. As shown in Table S3, among all patients carrying *WT1* c.1387C>T, 18 cases are male and the other 4 cases are female with highly variable presentations in clinic. Particularly, all female patients showed unilateral or bilateral Wilms’ tumor. Additionally, one woman showed ovarian dysgenesis and another had cysts in ovaries, indicating ovaries of 50% of the female patients were affected. By contrast, our proband did not exhibit any clinical features of Wilms’ tumor. And her POI symptoms were much milder compared to ovarian dysgenesis. So we reported an isolated POI patient carrying *WT1* c.1387C>T for the first time, suggesting that this pathogenic variant may only affect the function of ovaries during reproductive aging process.

Variable clinical features were also found in the male patients. Among the 18 cases, 2 (No. 21, 22) were diagnosed as Denys–Drash syndrome, 1 (No. 23) was diagnosed as Frasier syndrome, and 3 (No. 18, 19, 23) showed disorders of sex development without developing tumor. And the remaining 12 cases exhibited unilateral/bilateral Wilms’ tumor with or without nephrotic phenotypes/urinary tract malformations. It was hypothesized that the tumor tissues probably suffered a second hit during the gamete or embryonic stage due to specific factors, resulting in homozygous mutations or 11p13 loss of heterozygosity in the tumor (Cardoso et al., 2013). As to our male patient, the son of the proband, he was diagnosed with Wilms’ tumor and urethral malformation at 7 years of age without other symptoms, which was similar to several reported patients (No. 1, 2, 8, 9). Additionally, the variable clinical characteristics of patients carrying the same *WT1* variants might be related to gender, genetic background, environmental factors, and the mechanisms of *WT1* mutation, etc. All these findings are important for genetic counseling in clinic. And construction of *Wt1* knockout or knock‐in mice would be beneficial for determining the underlying pathogenic mechanism of *WT1* variants in POI.

Abbas et al. have found that mutant *WT1* mRNA transcripts that carry premature termination codons were sensitive to nonsense‐mediated RNA decay (NMD) in primary acute myeloid leukemia. According to the “50 bp rule”, *WT1* c.1387C>T, which is 61 bp upstream of the last exon–exon junction, may be likely to escape from NMD (Abbas et al., 2010). However, western blotting results using HEK293T cells further demonstrated that the c.1387C>T (p.R463*) variant could produce a truncated protein of WT1. The role of the truncated WT1 protein in ovarian development is of great value to be addressed in future work. *WT1* has been found to regulate apoptosis and proliferation of immature granulosa cells through regulation of the Wnt/β‐catenin signaling pathway (Y. Wang et al., 2019). These results would probably provide some insight into the subsequent specific functional assays to investigate the harmfulness of p.R463* altered WT1 protein.

Collectively, we report for the first time that a heterozygous c.1387C>T variant of *WT1* was associated with non–syndromic POI and Wilms’ tumor in a Chinese family. All our findings provide novel insight into the molecular mechanism of WT1 and genetic counseling for women with POI.

## CONFLICT OF INTEREST

The authors declare no conflict of interest.

## AUTHOR CONTRIBUTIONS

Xiaojin Zhang, Yanhua Wu, and Yingchen Wang designed the study. Yingchen Wang and Xiaojin Zhang performed clinical assessments. Qing Chen performed WES and data processing. Yingchen Wang, Xi Yang, Lingyue Shang, Yuncheng Pan, and Shuting Ren conducted Sanger sequencing, molecular cloning, and western blotting. Yingchen Wang, Xiaojin Zhang, Yanhua Wu, Qing Chen, Feng Zhang, Xi Yang, Zixue Zhou, Guoqing Li, Yunzheng Fang, and Li Jin analyzed the data. Yingchen Wang wrote the manuscript. Xiaojin Zhang and Yanhua Wu edited the manuscript. All authors confirmed the manuscript.

## 5 ETHICS STATEMENTS

The study was approved by the review boards of the Affiliated Obstetrics and Gynecology Hospital of Fudan University (Grant nos. 2017–19). Written informed consent was obtained from the patients and their families through interviews.

## Supporting information

Table S1‐S3‐Fig S1
