# Supplementary material for: Whole exome sequencing identified a rare WT1 loss‐of‐function variant in a non‐syndromic POI patient
Source: Mol Genet Genomic Med. 2021 Nov 29;10(1):e1820. doi: 10.1002/mgg3.1820 (PMC8801142; doi:10.1002/mgg3.1820)
Supplement: Supplementary file 1 — Table S1‐S3‐Fig S1 [file MGG3-10-e1820-s001.docx]

**Supplementary table 1** Representative data of the urinary testing of the POI subject affected by *WT1* variant.

| **Characteristic** | **Subject** | **Reference range** |
| --- | --- | --- |
| BUN (mmol/l) | 3.8 | 2.5~6.4 |
| Uretic Acid (μmol/l) | 288 | 155~357 |
| Creatine (μmol/l) | 48 | 40~66 |
| eGFR (ml/min/1.73m^2^) | 158 | ≥90 |
| Urine specific gravity | 1.020 | 1.003~1.030 |
| Urine pH | 6.5 | 6.0~7.0 |
| Urine leukocyte esterase | negative | negative |
| Urine nitrite | negative | negative |
| Urine protein | negative | negative |
| Urine glucose | negative | negative |
| Urine ketones | negative | negative |
| Urobilinogen | weakly positive | weakly positive |
| Urine bilirubin | negative | negative |
| Urine occult blood | negative | negative |
| Urine color | yellow |  |
| Urine transparency | slightly turbid |  |
| Microscopic examination of WBC | not seen | 0-5 |
| Microscopic examination of RBC | not seen | 0-3 |

BUN blood urea nitrogen, eGFR estimated glomerular filtration rate, WBC white blood cell, RBC red blood cell

**Supplementary table 2** Representative data of the thyroid testing of the POI subject affected by *WT1* variant.

| **Characteristic** | **Subject** | **Reference range** |
| --- | --- | --- |
| TT4 (nmol/l) | 133.70 | 66~181 |
| TT3 (nmol/l) | 2.09 | 1.3~3.1 |
| FT4 (pmol/l) | 17.68 | 12.00~22.00 |
| FT3 (pmol/l) | 5.25 | 3.10~6.80 |
| hCT (pg/ml) | 1.08 | <6.40 |
| Anti-TPO (IU/ml) | 14.21 | <34.00 |
| Anti-TSHR (IU/l) | <0.30 | <1.75 |
| Anti-Tg (IU/ml) | 17.57 | <115 |

TT4 total thyroxine, TT3 total triiodothyronine, FT4 free thyroxine, FT3 free triiodothyronine, hCT human calcitonin, Anti-TPO anti-thyroid peroxidase, anti-TSHR Anti-thyroid stimulating hormone receptor, Anti-Tg anti-thyroglobulin

**Supplementary table 3** Clinical features of patients carrying p.R463* in *WT1*

| **No.** | **Gender** | **Age** | **Phenotypes** | | | | | **Genetic alterations in tumor** | **Reference** |
| --- | --- | --- | --- | --- | --- | --- | --- | --- | --- |
|  |  |  | **Wilms’**  **tumor** | **Anomalies in genitourinary** | **Anomalies in gonads development** | **Nephropathy symptoms** | **Others** |  |  |
| 1 | 46,XY | 12m | Bilateral | - | - | - | - | Homozygosity | (Kaneko et al., 2015) |
| 2 | 46,XY | 9m | Bilateral | Hypospadias; Cryptorchidism | - | - | - | Homozygosity |  |
| 3 | 46,XY | 1y4m | Bilateral | Hypospadias | - | - | - | - |  |
| 4 | 46,XY | 1y1m | Bilateral | - | - | - | - | Homozygosity |  |
| 5 | 46,XX | 7m | Bilateral | - | Ovarian dysgenesis | Focal glomerular sclerosis | - | Homozygosity |  |
| 6 | 46,XX | 1y7m | Bilateral | - | - | - | - | Homozygosity |  |
| 7 | 46,XY | 2y | Unilateral | - | Maldescended testes | Proteinuria | - | - | (Schumacher et al., 1997) |
| 8 | 46,XY | 1y | Unilateral | Developmental structural anomalies | - | - | - | - | (Royer-Pokora et al., 2004) |
| 9 | 46,XY | 1y5m | Unilateral | Developmental structural anomalies | - | - | - | - |  |
| 10 | 46,XY | 3y11m | Unilateral | Unknown | - | - | - | - |  |
| 11 | 46,XY | 9m | Bilateral | Cryptorchidism | - | - | - | Homozygosity | (Shibata et al., 2002) |
| 12 | 46,XY | 11m | Bilateral | Hypospadias; Cryptorchidism | - | - | - | Homozygosity |  |
| 13 | 46,XY | 1y | Bilateral | Cryptorchidism | - | - | - | LOH |  |
| 14 | 46,XY | 1y | Unilateral | - | - | - | - | LOH |  |
| 15 | 46,XY | 11m | Bilateral | - | - | - | - | LOH | (Little et al., 1992) |
| 16^2^ | 46,XY | NA | Bilateral | Micropenis;  cryptorchidism | Partial  gonadal dysgenesis | ‘Drash’ nephropathy | Gross motor delay; Craniostenosis;  Prominent metopic suture;  Horseshoe kidney | Homozygosity | (Little et al., 1993) |
| 17 | 46,XY | 29y | - | Hypospadias; Normal phallus;  Hypoplastic scrotum;  Bilateral cryptorchidism  (at birth); Rudimentary uterus and proximal vaginal rest | Abdominal hypoplastic testes (18m);  Hypoplastic testes (27y) | Proteinuria (12y);  Focal segmental  glomerulosclerosis;  Arterial hypertension (18y) | - | - | (Kohler et al., 2011) |
| 18 | 46,XY | 18.5y | - | Penile hypospadias; Hypoplastic scrotum;  Bilateral abdominal cryptorchidism; Rudimentary uterus and vaginal rest; Hypoplastic testes | Disorder of sex development | Focal segmental glomerulosclerosis;  Proteinuria | - | - | (Lipska et al., 2013) |
| 19 | 46,XX | 15.3y | Wilms’ tumor (0.8y) | - | Ovarian cyst | Focal segmental glomerulosclerosis;  Proteinuria | - | - |  |
| 20 | 46,XY | 25m | Unilateral | - | - | Proteinuria | - | Heterozygosity;  A synonymous variant p.R369R (rs16754) | (Cardoso et al., 2013) |
| 21^1^ | 46,XX | 28m | Unilateral | - | - | Proteinuria (28m);  Diffuse mesangial sclerosis | - | - | (Yue et al., 2011) |
| 22^2^ | 46,XY | 25y | - | Hypospadias;  Cryptorchidism | Pseudoharmaphroditism with retentio testis | Proteinuria (8y);  Global glomerulosclerosis (16y);  Renal insufficiency (19y) | Gonadoblastoma | - | (Kohsaka et al., 1999) |

NA not available, LOH loss of heterozygosity, m month, y year

^1^ Case 16 was definitely diagnosed as Denys-Drash syndrome, and case 21 was definitely diagnosed as incomplete Denys-Drash syndrome.

^2^ Case 22 was definitely diagnosed as Frasier syndrome.


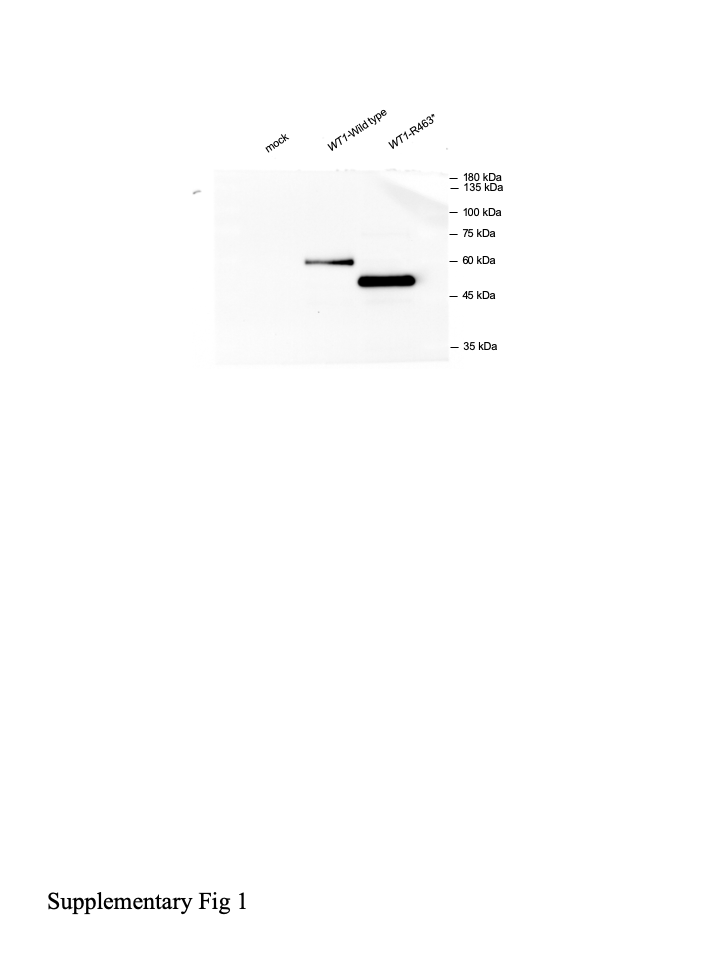


**Supplementary figure** Non-cropped picture of western blot
